# Normative reference data for body composition in healthy indigenous populations on the Qinghai-Tibet Plateau

**DOI:** 10.3389/fpubh.2022.986720

**Published:** 2022-10-06

**Authors:** Wenhui Li, Xin Li, Ting Huang, Ying Chen, Hailong Zhang, Hongliang Dai, Youfeng Wen

**Affiliations:** ^1^Experimental Teaching Center of Basic Medicine, Jinzhou Medical University, Jinzhou, China; ^2^Institute of Biological Anthropology, Jinzhou Medical University, Jinzhou, China; ^3^School of Public Health, Jinzhou Medical University, Jinzhou, China; ^4^School of Nursing, Jinzhou Medical University, Jinzhou, China

**Keywords:** body composition, indigenous population, Qinghai-Tibet Plateau, Tibetan, healthy adults

## Abstract

Body composition represents an important aspect of physical fitness and has received increasing attention in recent years. The present study was designed and conducted to provide reference values for age-, sex-, and altitude-specific body composition for healthy indigenous Tibetan adults living on the Qinghai-Tibet Plateau, which has not yet been studied. A cross-sectional survey was conducted among 2,356 healthy Tibetan adults (1,433 females and 923 males) living on the Qinghai-Tibet Plateau for generations. The body composition of the participants was measured by bioelectrical impedance analysis (BIA). The measurements included weight, basal metabolic rate (BMR), fat-free mass (FFM), skeletal muscle mass (SMM), protein mass (PM), bone mass (BM), fat mass (FM), percentage of body fat (PBF), subcutaneous fat mass (SFM), visceral fat mass (VFM), total body water (TBW), intracellular water (ICW), and extracellular water (ECW). Overall, males had greater FFM, SMM, PM, BM, and water mass, whereas females were high in fat mass. Participants from plains generally had higher body composition values, and PM, BM, FM and body water values decreased with increasing altitude, with the lowest values found in >4,000 m Shigatse. FFM, SMM, PM, and BM tended to decrease with increasing age, whereas fat mass and ECW tended to increase with increasing age. This study provides the first normative reference data of body composition for healthy indigenous individuals on the Qinghai-Tibet Plateau. These datasets are important for future research related to body composition. A considerable decrease in body composition values in > 4,000 m-altitude areas suggested that body composition cutoff values should be established by altitude. Age-, sex-, and altitude-specific alterations in body composition values also inform the prevention and amendment of abnormal body composition changes and resultant health complications.

## Introduction

As one of the important branches of human biology, body composition research focuses on the level, organizational rules, measurement techniques, and associated factors of body composition, including fat, protein, water, carbohydrates, and inorganic salts at the molecular level (1–3), and body size and configuration, which are generally referred to as anthropometric measures, such as body weight, skinfold thickness, and body mass index (BMI) at the whole-body level (3, 4). Body composition measurement is helpful for learning about overall health and nutrition status and provides meaningful information for disease diagnosis and treatment (5, 6). A large number of studies have found that changes in body composition are associated with a plethora of diseases, such as diabetes (7), hypertension (8), obesity (9), and cancer (10). Approximately 10–20% of cancer deaths are thought to be caused by malnutrition. Sarcopenia, a condition with loss of muscle mass and function, predicts a greater incidence of postsurgical complications (11). In addition, excessive visceral fat was associated with an increased incidence of chronic health complications, such as hypertension and diabetes (12, 13). Given the practical importance of body composition data, increasing research has focused on this subject in the past few years (14, 15).

Body composition is affected by many factors, including environment, genetics, sex, age, ethnicity, and altitude (16–20). Studies have shown that visceral fat increases with aging, particularly beyond 40 years of age (21). Additionally, intra-abdominal fat was documented to be increased in weight-stable elderly individuals in Hong Kong, China (22). Extracellular water (ECW)/total body water (TBW), an important index for the determination of malnutrition and dehydration, has been shown to be upregulated with aging in both sexes, with a greater amplitude of increase seen in males than in females (16, 23). The between-ethnicity difference in body composition has also been frequently reported. For instance, evidence showed that non-Hispanic blacks had lower cutoffs for the percentage of body fat than non-Hispanic whites and Mexican Americans (24). According to a recent systematic review, mountaineering stays of 14 days or more at altitudes >4,000 m produce decreases in total mass, fat mass, and lean mass (25). Consistent with this, our recent study found that among those aged 51–60 years, the incidence of sarcopenia in 4,200 m-altitude Shigatse was strikingly higher than that in 3,600 m-altitude Lhasa (32.9 vs. 3.5%) (26). However, the contribution of altitude alone to body composition change remains inconclusive due to the methodological limitations of the heterogeneity of the included studies (25). Based on these arguments, determination of normative reference values of body composition within a specific subpopulation is the premise for practical and clinical application of this biological index. To the authors' knowledge, body composition research in general healthy indigenous populations living in high-altitude regions, such as the Qinghai-Tibet Plateau, is scarce, although many extant body composition studies have focused attention on specific populations, such as elderly individuals, children, athletes, and obese individuals (3, 27–30). Moreover, although a recent study analyzed age- and sex-related differences among healthy subjects, the sample population used was Han adults (aged 18–82 years) living in the low-altitude Shannxi Province (16).

This study aims to investigate the range of body composition of indigenous Tibetan people living on the Qinghai-Tibet Plateau stratified by sex, age, and altitude to establish a reference database of body composition standards of indigenous plateau populations. This work will help provide relevant baseline data for anthropology, medicine and other disciplines. These data also provide a basis for evaluating the physical fitness of aboriginal people in high plateau regions.

## Materials and methods

### Participants

The survey was carried out through a health examination on the high-altitude area of Qinghai-Tibet Plateau and the low-altitude area of Jinzhou in China. A total of 2,356 healthy Tibetan adults (1,433 females and 923 males) aged 20–84 years old (43.29 ± 13.95 years on average) from Lhasa (altitude of 3,600 m) and Shigatse (altitude of 4,200 m) participated in this study. A total of 1,376 of the respondents were from Lhasa (812 females and 564 males), and 980 were from Shigatse (621 females and 359 males). All Tibetan participants had lived for generations in Tibet, China. To determine the influence of altitude on body composition variations, 748 healthy participants (493 females and 255 males) aged 20–87 years were enrolled from Jinzhou (altitude of 25 m) and served as a control population. Individuals with acute diseases, severe heart, lung, liver, kidney or brain dysfunctions, inflammatory reaction diseases, malignant tumors, endocrine diseases and pregnant women were excluded from this survey.

### Assessment of body composition

An MC-180 body composition analyzer (Tanita, Japan) was used to measure human body composition. During the measurement, subjects stood barefoot on the platform of the device, with their feet on the electrodes on both sides. They then grasped the handle of the device with their hands maintaining direct contact with the electrodes and their arms hanging naturally at their sides. The participants remained stationary for ~1 min during the measurement process. Metal articles such as ornaments, wrist watches, and cell phones were removed from the subjects. Specific recorded measurements included weight, basal metabolic rate (BMR), fat-free mass (FFM), skeletal muscle mass (SMM), protein mass (PM), bone mass (BM), fat mass (FM), percentage of body fat (PBF), subcutaneous fat mass (SFM), visceral fat mass (VFM), total body water (TBW), intracellular water (ICW), and extracellular water (ECW).

A portable stadiometer (HM200P, American Charder Company, USA) was used to measure height, which was recorded to the nearest 0.01 cm. Body mass index (BMI) was calculated from height (m) and weight (kg): BMI = weight (kg)/height (m^2^).

### Statistical analysis

The data are presented as the means ± standard deviations (SDs). Sex differences within the same age group were compared using an independent samples *t*-test. Analysis of variance (ANOVA) was used to test for differences between age groups and region groups. All analyses were performed using SPSS (ver. 21.0, IBM Company), and *P* < 0.05 was considered statistically significant.

## Results

### Whole-body composition measurements stratified by sex, altitude, and age

Whole-body composition variables measured by height, weight, BMI, and BMR are shown in Table 1 and stratified by sex, altitude, and age. According to the data, compared with females, males had significantly higher height, weight, and BMR (*P* < 0.05), regardless of stratification by altitude or age. Compared with those from Shigatse, participants from Lhasa and Jinzhou had significantly higher height, weight, BMI, and BMR (*P* < 0.05). This phenomenon was consistently seen in both sexes and all age groups. A downward trend of height and BMR was found with increased age (*P* < 0.05), whereas an upward trend was found in BMI with increasing age (*P* < 0.05).

**Table 1:** Sex-specific whole-body composition measurements stratified by age and altitude.

**Sex**	**Region**	**Age (years)**	***n***	**Height (cm)**	**Weight (kg)**	**BMI (kg/m^2^)**	**BMR (kcal/d)**
Females	Lhasa	20~	176	160.79 ± 4.99^#^^✰^^▴^	55.94 ± 9.84^#^	21.59 ± 3.37^#^^▴^	1,210.63 ± 128.73^#^^▴^
		31~	134	159.82 ± 5.14^#^^✰^^▴^	61.42 ± 11.46^#Δ▴^	23.98 ± 3.99^#✰Δ^	1,239.57 ± 141.14^#▴^
		41~	225	159.46 ± 5.23^#Δ▴^	63.81 ± 11.26^#Δ▴^	25.04 ± 4.00^#✰Δ^	1,243.08 ± 138.28^#Δ▴^
		51~	174	157.66 ± 5.83^#Δ▴^	62.55 ± 10.11^#Δ▴^	25.16 ± 3.84^#Δ^	1,190.39 ± 123.68^#▴^
		61~84	103	153.40 ± 5.97^#✰Δ^	57.15 ± 9.51^#✰^	24.24 ± 3.62^#Δ^	1,081.83 ± 118.51^#✰Δ^
		Mean	812	158.65 ± 5.84^#^	60.60 ± 10.98^#^	24.04 ± 4.02^#^	1,203.72 ± 140.47^#^
	Shigatse	20~	138	154.72 ± 4.79^*✰▴^	49.46 ± 7.35^*✰^	20.64 ± 2.55^*✰^	1,131.26 ± 98.20^*✰▴^
		31~	144	154.04 ± 4.79^*✰▴^	50.30 ± 6.30^*✰▴^	21.21 ± 2.31^*✰^	1,105.45 ± 88.16^*✰Δ▴^
		41~	145	154.31 ± 5.12^*✰▴^	52.76 ± 7.21^*✰Δ▴^	22.14 ± 2.66^*✰Δ▴^	1,113.45 ± 101.50^*✰▴^
		51~	124	152.10 ± 5.25^*✰Δ▴^	50.79 ± 7.48^*✰▴^	21.91 ± 2.93^*✰Δ^	1,047.88 ± 99.14^*✰Δ▴^
		61~79	70	149.38 ± 5.29^*✰Δ^	47.61 ± 6.89^*✰^	21.27 ± 2.55^*✰^	980.01 ± 95.72^*✰Δ^
		Mean	621	153.34 ± 5.27^*✰^	50.48 ± 7.21^*✰^	21.45 ± 2.66^*✰^	1,087.42 ± 107.53^*✰^
	Jinzhou	20~	110	162.17 ± 5.80^*#▴^	58.69 ± 13.76^#^	22.22 ± 4.33^#▴^	1,240.05 ± 201.28^#▴^
		31~	104	161.35 ± 4.93^*#▴^	59.43 ± 9.33^#^	22.76 ± 2.95^*#▴^	1,232.00 ± 149.46^#▴^
		41~	88	160.24 ± 5.07 ^Δ#▴^	61.69 ± 9.85^#^	23.99 ± 3.45^*#Δ▴^	1,236.30 ± 122.18^#▴^
		51~	72	157.67 ± 5.05 ^Δ#▴^	61.12 ± 8.12^#^	24.59 ± 3.19^#Δ^	1,193.75 ± 107.23^#▴^
		61~87	119	155.38 ± 5.69^*Δ#^	60.64 ± 8.57^*#^	25.00 ± 2.92^#Δ^	1,145.50 ± 120.71^*#Δ^
		Mean	493	159.35 ± 5.94^#^	60.21 ± 10.29^#^	23.67 ± 3.58^#^	1,208.10 ± 151.38^#^
Males	Lhasa	20~	126	172.92 ± 6.01^#▴※^	67.23 ± 13.23^#※^	22.48 ± 4.34^#^	1,543.98 ± 204.49^#▴※^
		31~	98	171.28 ± 5.65^#✰Δ▴※^	69.72 ± 11.54^#✰※^	23.72 ± 3.43^#^	1,519.92 ± 184.15^#✰▴※^
		41~	149	169.47 ± 6.50^#✰Δ▴※^	68.02 ± 12.70^#✰※^	23.65 ± 4.04^#✰※^	1,463.88 ± 201.42^#✰Δ▴※^
		51~	120	168.25 ± 5.48^#Δ▴※^	67.03 ± 12.20^#✰※^	23.60 ± 3.66^✰※^	1,418.22 ± 195.32^#✰Δ▴※^
		61~81	71	165.36 ± 6.67^#✰Δ※^	65.28 ± 9.78^#✰※^	23.83 ± 3.07^#✰^	1,352.27 ± 165.21^#✰Δ※^
		Mean	564	169.78 ± 6.49^#※^	67.58 ± 12.21^#✰※^	23.41 ± 3.84^#✰※^	1,467.74 ± 203.06^#✰※^
	Shigatse	20~	96	166.56 ± 5.80^*✰▴※^	55.89 ± 7.77^*✰※^	20.10 ± 2.56^*✰^	1,378.82 ± 140.90^*✰▴※^
		31~	104	165.06 ± 5.72^*✰▴※^	58.99 ± 8.76^*✰※^	21.65 ± 2.67^*✰Δ^	1,353.63 ± 141.90^*✰▴※^
		41~	73	166.03 ± 6.66^*✰▴※^	59.29 ± 8.84^*✰※^	21.45 ± 2.66^*✰Δ※^	1,337.77 ± 161.48^*✰▴※^
		51~	53	165.73 ± 6.43^*✰▴※^	61.36 ± 11.65^*✰Δ▴※^	22.42 ± 4.10^✰Δ^	1,319.70 ± 182.12^*✰▴※^
		61~77	33	161.63 ± 6.03^*✰Δ※^	54.82 ± 7.72^*✰※^	20.87 ± 2.78^*✰^	1,183.61 ± 131.59^*✰Δ※^
		Mean	359	165.44 ± 6.19^*✰※^	58.19 ± 9.12^*✰※^	21.24 ± 3.00^*✰^	1,336.50 ± 159.48^*✰※^
	Jinzhou	20~	42	173.38 ± 6.85^#▴※^	72.07 ± 17.55^#※^	23.75 ± 4.57^#^	1,583.86 ± 269.83^#※^
		31~	34	174.29 ± 3.51^*#▴※^	75.54 ± 9.87^*#▴※^	24.81 ± 2.89^#※^	1,645.71 ± 141.59^*#▴※^
		41~	60	172.03 ± 6.25^*#▴※^	77.21 ± 9.68^*#▴※^	26.09 ± 3.13^*#※^	1,657.93 ± 153.75^*#▴※^
		51~	59	168.75 ± 5.21^Δ#※^	70.78 ± 9.45^*#※^	24.85 ± 3.03^*#^	1,525.90 ± 157.88^*#※^
		61~81	60	167.46 ± 7.35 ^*#Δ※^	71.47 ± 10.36^*#※^	25.46 ± 2.81^*#^	1,468.45 ± 212.39^*#※^
		Mean	255	170.72 ± 6.61^#※^	73.30 ± 11.67^*#※^	25.10 ± 3.35^*#※^	1,568.97 ± 203.79^*#※^

Data are presented as the means ± SDs. Compared with Lhasa for the same age and sex,

* P < 0.05; compared with Shigatse for the same age and sex,

# P < 0.05; compared with Jinzhou for the same age and sex,

✰ P < 0.05; compared with the 20~ age group for the same altitude and sex,

Δ P < 0.05; compared with the 60~ age group for the same altitude and sex,

▴ P < 0.05; compared with females for the same altitude and age,

※ P < 0.05.

BMI, body mass index; BMR, basal metabolic rate.

### Body composition of protein content stratified by sex, altitude, and age

Protein mass represented by FFM, SMM, PM, and PP stratified by sex, altitude, and age are shown in Table 2. From these data, in all three investigation regions, males had significantly higher FFM, SMM, PM, and PP values than females across all age groups (*P* < 0.05). Respondents from Jinzhou had the highest FFM, SMM, and PM values, followed by those from Lhasa, and the lowest values were found among those in high-altitude Shigatse, after controlling for sex and age (*P* < 0.05). However, participants from Shigatse had the largest PP value among participants from these three altitudes. The maximum FFM and SMM values occurred in the 41~ age group in these three regions for females and for males in Shigatse. The PM values of women decreased with age. For men from Lhasa and Jinzhou, the maximum FFM, SMM, and PM values mostly occurred in the 31~ age group. The 61~ age group had the lowest FFM, SMM, and PM values after controlling for altitude and sex, which were significantly lower than those among young and middle-aged people (*P* < 0.05).

**Table 2:** Sex-specific body composition of protein mass as stratified by age and altitude.

**Sex**	**Region**	**Age (years)**	***n***	**FFM (kg)**	**SMM (kg)**	**PM (kg)**	**PP (%)**
Females	Lhasa	20~	176	39.62 ± 3.67^#▴^	37.32 ± 3.36^#▴^	9.39 ± 0.95^#▴^	17.22 ± 2.97^▴^
		31~	134	41.20 ± 3.82^#Δ▴^	38.75 ± 3.49^#Δ▴^	8.84 ± 1.13^#✰Δ▴^	14.94 ± 3.49^#✰Δ▴^
		41~	225	41.70 ± 3.80^#Δ▴^	39.22 ± 3.47^#Δ▴^	8.47 ± 1.10^#Δ▴^	13.78 ± 3.30^#Δ^
		51~	174	40.15 ± 3.52^#▴^	37.80 ± 3.22^#▴^	8.21 ± 1.20^#Δ▴^	13.56 ± 3.20^#Δ^
		61~84	103	37.02 ± 3.44^#✰Δ^	34.94 ± 3.14^#✰Δ^	7.63 ± 1.09^Δ^	13.77 ± 3.20^#Δ^
		Mean	812	40.24 ± 3.95^#^	37.88 ± 3.60^#^	8.57 ± 1.22^#^	14.67 ± 3.52^#^
	Shigatse	20~	138	37.65 ± 3.01^*✰▴^	35.50 ± 2.75^*✰▴^	8.27 ± 0.82^*✰▴^	17.03 ± 2.62^▴^
		31~	144	37.76 ± 2.85^*✰▴^	35.61 ± 2.60^*✰▴^	8.13 ± 0.81^*✰▴^	16.37 ± 2.34^*Δ^
		41~	145	38.45 ± 3.27^*✰▴^	36.25 ± 2.99^*✰Δ▴^	7.93 ± 0.94^*✰Δ▴^	15.31 ± 2.69^*Δ^
		51~	124	36.48 ± 3.19^*✰Δ▴^	34.44 ± 2.91^*✰Δ▴^	7.78 ± 0.95^*✰Δ▴^	15.62 ± 2.73^*✰Δ^
		61~79	70	34.48 ± 3.05^*✰Δ^	32.62 ± 2.79^*✰Δ^	7.42 ± 0.85^✰Δ^	15.87 ± 2.61^*✰Δ^
		Mean	621	37.27 ± 3.29^*✰^	35.17 ± 3.01^*✰^	7.97 ± 0.91^*✰^	16.06 ± 2.67^*✰^
	Jinzhou	20~	110	40.53 ± 6.30^#^	38.18 ± 5.92^#^	9.65 ± 1.55^#▴^	17.05 ± 3.59^▴^
		31~	104	41.40 ± 4.80^#▴^	38.97 ± 4.48^#▴^	9.34 ± 1.50^*#▴^	16.03 ± 2.94^*▴^
		41~	88	41.92 ± 3.37^#▴^	39.41 ± 3.09^#▴^	8.70 ± 1.26^#Δ▴^	14.52 ± 3.22^Δ▴^
		51~	72	40.71 ± 3.15^#▴^	38.32 ± 2.89^#▴^	8.25 ± 1.15^#Δ▴^	13.82 ± 3.01^#Δ^
		61~87	119	39.09 ± 3.75^*#^	36.83 ± 3.49^*#^	7.87 ± 1.36^#Δ^	13.24 ± 2.82^#Δ^
		Mean	493	40.64 ± 4.63^#^	38.26 ± 4.31^#^	8.78 ± 1.55^#^	14.99 ± 3.43^#^
Males	Lhasa	20~	126	54.01 ± 6.64^#▴※^	51.21 ± 6.31^#▴※^	15.28 ± 2.49^#▴※^	22.94 ± 2.01^#▴※^
		31~	98	54.26 ± 5.86^#✰▴※^	51.45 ± 5.56^#✰▴※^	15.79 ± 2.19^#▴※^	22.80 ± 1.95^#✰▴※^
		41~	149	53.11 ± 6.42^#✰▴※^	50.34 ± 6.10^#✰▴※^	14.76 ± 2.22^#✰▴※^	21.96 ± 2.43^✰Δ▴※^
		51~	120	51.90 ± 6.32^#✰Δ▴※^	49.20 ± 6.02^#✰Δ▴※^	14.42 ± 2.06^#Δ▴※^	21.75 ± 2.17^✰Δ▴※^
		61~	71	49.97 ± 5.54^#✰Δ※^	47.37 ± 5.26^#✰Δ※^	13.42 ± 2.20^#✰Δ※^	20.64 ± 2.23^#Δ※^
		Mean	564	52.86 ± 6.38^#✰※^	50.11 ± 6.06^#✰※^	14.81 ± 2.34^#※^	22.11 ± 2.30^#✰※^
	Shigatse	20~	96	48.84 ± 4.79^*✰▴※^	46.29 ± 4.55^*✰▴※^	12.08 ± 1.71^*✰※^	21.70 ± 2.03^*✰※^
		31~	104	48.94 ± 4.69^*✰▴※^	46.39 ± 4.46^*✰▴※^	12.82 ± 2.19^*✰※^	21.73 ± 1.97^*※^
		41~	73	49.08 ± 5.50^*✰▴※^	46.53 ± 5.22^*✰▴※^	13.09 ± 1.93^*✰Δ▴※^	22.16 ± 1.95^✰※^
		51~	53	48.66 ± 6.08^*✰▴※^	46.11 ± 5.77^*✰▴※^	12.96 ± 2.30^*✰※^	21.25 ± 2.23^✰※^
		61~	33	44.31 ± 4.57^*✰Δ※^	41.99 ± 4.34^*✰Δ※^	11.85 ± 1.62^*✰※^	21.75 ± 2.56^※^
		Mean	359	48.47 ± 5.25^*✰※^	45.94 ± 4.98^*✰※^	12.61 ± 2.03^*✰※^	21.74 ± 2.09^*✰※^
	Jinzhou	20~	42	55.16 ± 8.78^#※^	52.28 ± 8.37^#※^	16.41 ± 3.81^#▴※^	22.94 ± 2.40^#▴※^
		31~	34	58.74 ± 4.15^*#Δ▴※^	57.71 ± 3.96 ^*#Δ▴※^	16.45 ± 1.79^#▴※^	21.88 ± 1.52^*Δ▴※^
		41~	60	59.57 ± 4.82^*#▴※^	56.49 ± 4.58^*#▴※^	15.35 ± 2.24^*#▴※^	19.99 ± 2.44^*#Δ※^
		51~	59	55.58 ± 5.22^*#※^	52.69 ± 4.97^*#※^	14.18 ± 1.81^#Δ※^	20.19 ± 2.53^*#Δ※^
		61~	60	53.71 ± 6.99^*#※^	50.93 ± 6.65^*#※^	14.22 ± 2.15^*#Δ※^	20.04 ± 2.54^#Δ※^
		Mean	255	56.43 ± 6.55^*#※^	53.50 ± 6.24^*#※^	15.13 ± 2.59^#※^	20.78 ± 2.62^*#※^

Data are presented as the means ± SDs. Compared with Lhasa for the same age and sex,

* P < 0.05; compared with Shigatse for the same age and sex,

# P < 0.05; compared with Jinzhou for the same age and sex,

✰ P < 0.05; compared with the 20~ age group for the same altitude and sex,

Δ P < 0.05; compared with the 60~ age group for the same altitude and sex,

▴ P < 0.05; compared with females for the same altitude and age,

※ P < 0.05.

FFM, fat-free mass; SMM, skeletal muscle mass; PM, protein mass; PP, percentage of protein.

### Body composition of fat content stratified by sex, altitude, and age

Fat mass represented by FM, VFM, SFM, and PBF stratified by sex, altitude, and age are shown in Table 3. From these data, in the three investigation regions, females had significantly higher FM, SFM, and PBF values than males across all age groups (*P* < 0.05). For females, respondents from Jinzhou and Lhasa had significantly higher FM, VFM, SFM, and PBF values than those from high-altitude Shigatse after controlling for sex and age (*P* < 0.05). Among male respondents, the FM, VFM, SFM, and PBF values in Jinzhou were higher than those in Lhasa (*P* < 0.05), and these values in Lhasa were also higher than those in Shigatse (*P* < 0.05). The mean values of fat-related indexes tended to increase with age. Compared with the older age groups, the 20~ age group had significantly lower values after controlling for altitude and sex (*P* < 0.05).

**Table 3:** Sex-specific body composition of fat mass as stratified by age and altitude.

**Sex**	**Region**	**Age (years)**	***n***	**FM (kg)**	**VFM (kg)**	**SFM (kg)**	**PBF (%)**
Females	Lhasa	20~	176	16.33 ± 6.90^#▴^	1.66 ± 1.57^#▴^	14.66 ± 5.41^#▴^	28.18 ± 6.51^#▴^
		31~	134	20.25 ± 8.36^#Δ^	2.67 ± 1.90^#Δ▴^	17.56 ± 6.49^#Δ^	31.65 ± 7.80^#Δ▴^
		41~	225	22.14 ± 8.42^#✰Δ^	3.30 ± 2.07^#✰Δ^	18.82 ± 6.39^#✰Δ▴^	33.50 ± 7.52^#✰Δ^
		51~	174	22.43 ± 7.86^#✰Δ^	3.55 ± 1.99^#✰Δ^	18.87 ± 5.90^#✰Δ▴^	34.86 ± 7.22^#✰Δ^
		61~84	103	20.15 ± 7.10^#Δ^	3.35 ± 1.74^#Δ^	16.79 ± 5.41^#Δ^	34.19 ± 7.57^#Δ^
		Mean	812	20.38 ± 8.14^#^	2.90 ± 2.01^#^	17.46 ± 6.19^#^	32.42 ± 7.69^#^
	Shigatse	20~	138	11.84 ± 5.18^*✰^	0.86 ± 0.81^*✰▴^	10.96 ± 4.41^*✰^	23.07 ± 6.48^*✰▴^
		31~	144	12.57 ± 4.54^*✰^	1.05 ± 0.78^*✰▴^	11.50 ± 3.78^*✰^	24.37 ± 5.86^*✰^
		41~	145	14.33 ± 5.36^*✰Δ^	1.46 ± 1.03^*✰Δ^	12.85 ± 4.36^*✰Δ▴^	26.44 ± 6.56^*✰Δ^
		51~	124	14.33 ± 5.82^*✰Δ^	1.71 ± 1.26^*✰Δ^	12.61 ± 4.59^*✰Δ^	27.36 ± 7.21^*✰Δ^
		61~79	70	13.15 ± 4.96^*✰^	1.64 ± 1.01^*✰Δ^	11.50 ± 3.97^*✰^	26.87 ± 6.51^*✰Δ^
		Mean	621	13.23 ± 5.28^*✰^	1.30 ± 1.04^*✰^	11.92 ± 4.30^*✰^	25.44 ± 6.71^*✰^
	Jinzhou	20~	110	18.18 ± 8.38^#▴^	2.08 ± 1.87^#▴^	16.09 ± 6.58^#^	29.64 ± 7.25^#▴^
		31~	104	18.05 ± 5.62^#▴^	2.06 ± 1.14^#▴^	15.99 ± 4.51^#▴^	29.77 ± 5.09^#▴^
		41~	88	19.80 ± 7.56^*#^	2.63 ± 1.81^*#▴^	17.16 ± 5.78^*#^	31.19 ± 6.57^*#▴^
		51~	72	20.44 ± 5.99^*#^	2.99 ± 1.37^*#Δ▴^	17.42 ± 4.65^*#^	32.77 ± 5.81^*#Δ▴^
		61~87	119	21.58 ± 5.97^#Δ^	3.55 ± 1.48^#Δ^	18.02 ± 4.54^#^	34.97 ± 5.45^#Δ^
		Mean	493	19.59 ± 6.93^#^	2.66 ± 1.67^#^	16.92 ± 5.34^#^	31.69 ± 6.42^#^
Males	Lhasa	20~	126	13.25 ± 7.63^#※^	1.94 ± 1.74^#▴^	11.29 ± 5.91^#※^	18.42 ± 7.45^#▴※^
		31~	98	15.49 ± 6.58^#※^	2.55 ± 1.63^#Δ▴^	12.93 ± 4.98^#※^	21.40 ± 5.94^#Δ※^
		41~	149	14.95 ± 7.33^#※^	2.65 ± 1.92^#Δ▴※^	12.28 ± 5.42^#✰※^	20.95 ± 6.57^#Δ※^
		51~	120	15.16 ± 6.86^#※^	2.89 ± 1.89^Δ※^	12.25 ± 5.00^#※^	21.67 ± 6.36^Δ※^
		61~	71	15.33 ± 5.36^#✰※^	3.16 ± 1.52^#✰Δ^	12.15 ± 4.07^#✰※^	22.83 ± 5.89^#Δ※^
		Mean	564	14.76 ± 7.00^#✰※^	2.59 ± 1.82^#✰※^	12.15 ± 5.23^#✰✰※^	20.85 ± 6.68^#✰※^
	Shigatse	20~	96	7.08 ± 4.03^*✰▴※^	0.69 ± 0.77^*✰▴^	6.39 ± 3.28^*✰▴※^	12.10 ± 4.98^*✰▴※^
		31~	104	10.08 ± 5.06^*✰Δ※^	1.34 ± 1.07^*✰Δ※^	8.73 ± 4.01^*✰Δ※^	16.33 ± 6.11^*✰Δ※^
		41~	73	10.23 ± 4.60^*✰Δ※^	1.52 ± 1.07^*✰Δ^	8.69 ± 3.54^*✰Δ※^	16.68 ± 5.43^*✰Δ※^
		51~	53	12.72 ± 7.36^*Δ^	2.32 ± 1.91^Δ※^	10.39 ± 5.47^*Δ※^	19.48 ± 8.36^Δ※^
		61~	33	10.54 ± 4.50^*✰Δ※^	1.91 ± 1.10^*✰Δ^	8.62 ± 3.41^*✰Δ※^	18.64 ± 5.99^*✰Δ※^
		Mean	359	9.74 ± 5.38^*✰※^	1.40 ± 1.28^*✰^	8.33 ± 4.14^*✰※^	15.95 ± 6.58^*✰※^
	Jinzhou	20~	42	16.92 ± 9.49^#^	2.85 ± 2.38^#※^	14.09 ± 7.16^#^	21.86 ± 7.87^*#※^
		31~	34	16.82 ± 6.41^#^	2.97 ± 1.53^#※^	13.86 ± 4.89^#^	21.58 ± 5.77^#※^
		41~	60	17.66 ± 5.86^#※^	3.27 ± 1.50^#▴※^	14.37 ± 4.37^*#※^	22.28 ± 5.45^#▴※^
		51~	59	15.23 ± 5.41^▴※^	2.81 ± 1.36^▴^	12.41 ± 4.10^※^	20.99 ± 5.40^▴※^
		61~	60	17.76 ± 5.49^*#※^	3.81 ± 1.48^*#^	13.94 ± 4.07^*#※^	24.47 ± 5.29^#※^
		Mean	255	16.89 ± 6.52^*#※^	3.18 ± 1.68^*#※^	13.70 ± 4.90^*#※^	22.33 ± 6.00^*#※^

Data are presented as the means ± SDs. Compared with Lhasa for the same age and sex,

* P < 0.05; compared with Shigatse for the same age and sex,

# P < 0.05; compared with Jinzhou for the same age and sex,

✰ P < 0.05; compared with the 20~ age group for the same altitude and sex,

Δ P < 0.05; compared with the 60~ age group for the same altitude and sex,

▴ P < 0.05; compared with females for the same altitude and age,

※ P < 0.05.

FM, fat mass; VFM, visceral fat mass; SFM, subcutaneous fat mass; PBF, percentage of body fat.

### Body composition of bone mass stratified by sex, altitude, and age

The bone mass and percentage of bone minerals stratified by sex, altitude, and age are shown in Table 4. From these data, in the three regions, males had significantly higher bone mass than females across all age groups (*P* < 0.05). Meanwhile, respondents from Jinzhou had the highest bone mass values, followed by those from Lhasa, and the lowest values were found in highest-altitude Shigatse after controlling for sex and age (*P* < 0.05). Peak bone mass occurred in the 41~ age group, with the exception of that in Lhasa men who had a peak bone mass in their thirties. Compared with the younger age groups, the 61~ age group had the lowest bone mass after controlling for altitude and sex (*P* < 0.05). The percentage of bone minerals showed a general downward trend with increasing age, and the Shigatse population had the highest percentage of bone minerals (*P* < 0.05).

**Table 4:** Sex-specific body composition of bone mass as stratified by age and altitude.

**Sex**	**Region**	**Age (years)**	***n***	**BM (kg)**	**PBM (%)**
Females	Lhasa	20~	176	2.30 ± 0.32^#▴^	4.15 ± 0.34^#▴^
		31~	134	2.44 ± 0.34^#Δ▴^	4.02 ± 0.39^#Δ▴^
		41~	225	2.48 ± 0.33^#Δ▴^	3.94 ± 0.39^#✰Δ▴^
		51~	174	2.35 ± 0.31^#▴^	3.79 ± 0.42^#✰Δ▴^
		61~84	103	2.08 ± 0.30^#✰Δ^	3.67 ± 0.39^#Δ^
		Mean	812	2.36 ± 0.34^#^	3.93 ± 0.42^#^
	Shigatse	20~	138	2.13 ± 0.26^*✰▴^	4.33 ± 0.36^*✰▴^
		31~	144	2.14 ± 0.25^*✰▴^	4.28 ± 0.35^*✰▴^
		41~	145	2.20 ± 0.29^*✰Δ▴^	4.18 ± 0.40^*✰Δ▴^
		51~	124	2.03 ± 0.28^*✰Δ▴^	4.03 ± 0.44^*Δ^
		61~79	70	1.86 ± 0.27^*✰Δ^	3.93 ± 0.40^*✰Δ^
		Mean	621	2.10 ± 0.29^*✰^	4.18 ± 0.41^*✰^
	Jinzhou	20~	110	2.35 ± 0.41^#▴^	4.06 ± 0.36^#▴^
		31~	104	2.42 ± 0.34^#▴^	4.10 ± 0.28^#▴^
		41~	88	2.50 ± 0.29^#Δ▴^	4.10 ± 0.39^*#▴^
		51~	72	2.40 ± 0.26^#▴^	3.95 ± 0.33^*Δ▴^
		61~87	119	2.25 ± 0.32^*#^	3.73 ± 0.32^#Δ^
		Mean	493	2.38 ± 0.34^#^	3.98 ± 0.37^#^
Males	Lhasa	20~	126	2.80 ± 0.33^#▴※^	4.24 ± 0.41^#▴※^
		31~	98	2.81 ± 0.30^#✰▴※^	4.08 ± 0.32^#Δ^
		41~	149	2.76 ± 0.32^#✰▴※^	4.12 ± 0.36^#※^
		51~	120	2.70 ± 0.31^#✰Δ▴※^	4.08 ± 0.35^#Δ※^
		61~	71	2.60 ± 0.28^#✰Δ※^	4.01 ± 0.30^#✰Δ※^
		Mean	564	2.75 ± 0.32^#✰※^	4.12 ± 0.36^#✰※^
	Shigatse	20~	96	2.54 ± 0.25^*✰▴※^	4.58 ± 0.27^*✰▴※^
		31~	104	2.55 ± 0.23^*✰▴※^	4.36 ± 0.34^*✰Δ^
		41~	73	2.56 ± 0.28^*✰▴※^	4.34 ± 0.29^*✰Δ※^
		51~	53	2.55 ± 0.31^*✰▴※^	4.22 ± 0.44^*✰Δ※^
		61~	33	2.31 ± 0.24^*✰Δ※^	4.25 ± 0.32^*✰Δ※^
		Mean	359	2.53 ± 0.27^*✰※^	4.38 ± 0.35^*✰※^
	Jinzhou	20~	42	2.88 ± 0.42^#※^	4.09 ± 0.42^#^
		31~	34	3.04 ± 0.19 ^*#Δ▴※^	4.06 ± 0.32^#^
		41~	60	3.09 ± 0.24^*#▴※^	4.03 ± 0.31^#▴^
		51~	59	2.89 ± 0.25^*#▴※^	4.11 ± 0.28^#▴※^
		61~	60	2.79 ± 0.34^*#※^	3.92 ± 0.28^*#※^
		Mean	255	2.93 ± 0.32^*#※^	4.04 ± 0.33^*#※^

Data are presented as the means ± SDs. Compared with Lhasa for the same age and sex,

* P < 0.05; compared with Shigatse for the same age and sex,

# P < 0.05; compared with Jinzhou for the same age and sex,

✰ P < 0.05; compared with the 20~ age group for the same altitude and sex,

Δ P < 0.05; compared with the 60~ age group for the same altitude and sex,

▴ P < 0.05; compared with females for the same altitude and age,

※ P < 0.05.

BM, bone mass; PBM, percentage of bone minerals.

### Body composition of water mass stratified by sex, altitude, and age

Water mass represented by TBW, ICW, ECW, and PBW stratified by sex, altitude, and age are shown in Table 5. From these data, in the three regions, males had significantly higher TBW, ICW, ECW, and PBW values than females across all age groups (*P* < 0.05). In addition, respondents from Jinzhou had the highest TBW, ICW, and ECW values, followed by those from Lhasa, and the lowest values were found among those living in Shigatse after controlling for sex and age (*P* < 0.05). However, the PBW value was the largest in the Shigatse population (*P* < 0.05). TBW, ICW, and ECW values among females first increased and then decreased with age, with peak values appearing in the 41~ age group. In contrast, among males, TBW and ICW exhibited a downward trend with increasing age. In addition, the ECW values among women from the plains and men from the plateau increased with age. The 20~ age group had the lowest ECW values after controlling for altitude and sex, which were significantly lower than those in the middle-aged and elderly groups (*P* < 0.05).

**Table 5:** Sex-specific body composition of water mass as stratified by age and altitude.

**Sex**	**Region**	**Age (years)**	***n***	**TBW (kg)**	**ICW (kg)**	**ECW (kg)**	**PBW (%)**
Females	Lhasa	20~	176	27.97 ± 3.25^#^	16.89 ± 1.86^▴^	11.10 ± 1.52^#▴^	50.52 ± 3.89^#✰▴^
		31~	134	29.96 ± 3.60^#Δ▴^	17.66 ± 1.99^#Δ▴^	12.31 ± 1.81^#Δ^	49.46 ± 4.59^#Δ^
		41~	225	30.78 ± 3.65^#Δ▴^	17.83 ± 2.17^#Δ▴^	12.98 ± 1.71^#Δ^	48.86 ± 4.51^#✰Δ^
		51~	174	29.63 ± 3.20^#Δ▴^	16.71 ± 1.94^#✰▴^	12.94 ± 1.48^#Δ^	47.86 ± 4.08^#✰Δ^
		61~84	103	27.35 ± 2.99^#✰^	14.87 ± 1.81^#✰Δ^	12.51 ± 1.43^#✰Δ^	48.44 ± 4.51^#Δ^
		Mean	812	29.35 ± 3.60^#^	16.98 ± 2.19^#^	12.39 ± 1.76^#^	49.05 ± 4.39^#^
	Shigatse	20~	138	27.28 ± 2.62^*▴^	17.02 ± 1.65^▴^	10.28 ± 1.13^*✰▴^	55.64 ± 4.34^*✰▴^
		31~	144	27.52 ± 2.42^*✰▴^	16.86 ± 1.62^*✰▴^	10.69 ± 0.99^*✰Δ▴^	55.05 ± 3.99^*✰▴^
		41~	145	28.35 ± 2.86^*✰Δ▴^	17.03 ± 1.97^*✰▴^	11.34 ± 1.13^*✰Δ^	54.14 ± 4.48^*✰Δ^
		51~	124	26.69 ± 2.58^*✰▴^	15.49 ± 1.72^*✰Δ▴^	11.23 ± 1.14^*✰Δ^	53.06 ± 4.74^*✰Δ^
		61~79	70	25.23 ± 2.54^*✰Δ^	14.20 ± 1.76^*✰Δ^	11.05 ± 1.01^*✰Δ^	53.42 ± 4.28^*✰Δ^
		Mean	621	27.24 ± 2.76^*✰^	16.36 ± 2.00^*✰^	10.90 ± 1.16^*✰^	54.39 ± 4.46^*✰^
	Jinzhou	20~	110	28.57 ± 5.11	17.12 ± 3.12^▴^	11.47 ± 2.10^#▴^	49.32 ± 4.00^*#^
		31~	104	29.68 ± 3.97^#^	17.71 ± 2.54^#▴^	11.99 ± 1.50^#▴^	50.17 ± 2.89^#▴^
		41~	88	30.75 ± 3.34^#Δ▴^	18.09 ± 2.09^#Δ▴^	12.69 ± 1.45^#Δ^	50.26 ± 3.81^*#▴^
		51~	72	30.10 ± 3.14^#▴^	17.27 ± 2.21^*#▴^	12.85 ± 1.26^#Δ^	49.53 ± 3.52^*#▴^
		61~87	119	29.00 ± 3.17^*#^	15.94 ± 2.02 ^*#Δ^	13.08 ± 1.32^*#Δ^	48.14 ± 3.32^#^
		Mean	493	29.52 ± 3.94^#^	17.16 ± 2.56^#^	12.39 ± 1.60^#^	49.41 ± 3.60^#^
Males	Lhasa	20~	126	35.97 ± 4.75^#▴※^	22.01 ± 3.30^▴※^	14.00 ± 1.60^#▴※^	54.47 ± 6.69^#✰※^
		31~	98	35.71 ± 4.28^#✰▴※^	21.32 ± 2.99^#✰▴※^	14.41 ± 1.38^#✰Δ※^	51.81 ± 5.47^#Δ※^
		41~	149	35.64 ± 4.94^#✰▴※^	21.00 ± 3.51^#✰Δ▴※^	14.66 ± 1.54^#✰Δ※^	53.07 ± 5.82^#※^
		51~	120	34.83 ± 4.91^#✰※^	20.13 ± 3.53^✰Δ※^	14.72 ± 1.48^#✰Δ※^	52.58 ± 5.79^✰※^
		61~	71	33.99 ± 4.43^#✰Δ※^	19.17 ± 3.31^#✰Δ※^	14.86 ± 1.23^#✰Δ※^	52.58 ± 6.07^※^
		Mean	564	35.35 ± 4.75^#✰※^	20.87 ± 3.46^#✰※^	14.51 ± 1.50^#✰※^	53.00 ± 6.04^#※^
	Shigatse	20~	96	34.25 ± 3.82^*▴※^	21.26 ± 2.82^▴※^	13.01 ± 1.09^*✰※^	61.69 ± 5.21^*✰▴※^
		31~	104	33.61 ± 3.49^*✰▴※^	20.23 ± 2.56^*✰Δ▴※^	13.41 ± 1.04^*✰※^	57.66 ± 6.62^*✰Δ※^
		41~	73	33.48 ± 4.21^*✰▴※^	19.74 ± 3.07^*✰Δ▴※^	13.76 ± 1.22^*✰Δ※^	56.90 ± 5.63^*✰Δ※^
		51~	53	33.21 ± 4.54^*✰▴※^	19.19 ± 3.35^✰Δ▴※^	14.04 ± 1.31^*✰Δ※^	55.14 ± 7.95^Δ※^
		61~	33	30.18 ± 3.86^*✰Δ※^	16.68 ± 2.87^*✰Δ※^	13.53 ± 1.09^*✰※^	55.44 ± 6.07^✰Δ^
		Mean	359	33.38 ± 4.05^*✰※^	19.93 ± 3.13^*✰※^	13.48 ± 1.19^*✰※^	58.01 ± 6.67^*✰※^
	Jinzhou	20~	42	35.91 ± 5.03^※^	21.63 ± 3.17^※^	14.31 ± 1.91^#▴※^	51.19 ± 6.49^*#※^
		31~	34	39.29 ± 3.19^*#Δ▴※^	23.77 ± 2.39^*#Δ▴※^	15.56 ± 0.96^*#Δ※^	52.53 ± 5.34^#※^
		41~	60	41.18 ± 4.06^*#Δ▴※^	24.91 ± 3.00^*#Δ▴※^	16.28 ± 1.19^*#Δ※^	53.78 ± 5.43^#※^
		51~	59	38.56 ± 4.36 ^*#Δ▴※^	22.88 ± 3.17^*#▴※^	15.70 ± 1.29^*#Δ※^	54.78 ± 4.66^*Δ▴※^
		61~	60	36.74 ± 5.41^*#※^	20.99 ± 4.06^*#※^	15.79 ± 1.42^*#Δ※^	51.61 ± 4.46^#※^
		Mean	255	38.41 ± 4.90^*#※^	22.83 ± 3.57^*#※^	15.61 ± 1.51^*#※^	52.91 ± 5.37^#※^

Data are presented as the means ± SDs. Compared with Lhasa for the same age and sex,

* P < 0.05; compared with Shigatse for the same age and sex,

# P < 0.05; compared with Jinzhou for the same age and sex,

✰ P < 0.05; compared with the 20~ age group for the same altitude and sex,

Δ P < 0.05; compared with the 60~ age group for the same altitude and sex,

▴ P < 0.05; compared with females for the same altitude and age,

※ P < 0.05.

TBW, total body water; ICW, intracellular water; ECW, extracellular water; PBW, percentage of body water.

## Discussion

As one of the three major plateaus in the world, the Qinghai-Tibet Plateau is a natural laboratory for studying the plateau environment. Tibetans have lived on the Qinghai-Tibet Plateau for generations. Their genes, metabolic level and intestinal flora have changed adaptively. These changes, together with their unique dietary habits and lifestyle, render them fully adapted to the living environment of the plateau. Lhasa (3,600 m above sea level) and Shigatse (4,200 m above sea level) are the permanent settlements of Tibetans. Through the comparative analysis of the body composition of Tibetans in the two regions, it was found that the reference value of each body composition index of Tibetans in Shigatse was lower than that of Tibetans in Lhasa, which was in line with previous reports showing that increased altitude reduced the body composition of the human body (25, 31). This influence of altitude on body composition changes was also corroborated by the highest level of reference values among participants from Jinzhou, the lowest-altitude (25 m above sea level) area in the current study. This difference may be related to the different living environments and economic levels of these regions. With the increase in altitude, the level of circulatory leptin increases, which promotes physical consumption, reduces food intake and affects the acquisition of FM (32). A high-altitude environment also undermines fat deposition by reducing abdominal fat and intramuscular lipids (33). Meanwhile, chronic hypoxia caused by high altitude accelerates the decomposition of skeletal muscle and inhibits protein synthesis, leading to the decline in SMM and TBW (34). In addition, long-term living in a hypoxic environment results in a decrease in bone turnover and BM (35). Regarding socioeconomic status, Tibetans in Lhasa are less engaged in traditional animal husbandry; in other words, they are less involved in physical labor than those from Shigatse, whereas a comparatively better socioeconomic status leads to more intake of a high-calorie diet in Lhasa. These differences would inevitably lead to an increase in body composition indexes, especially those related to fat mass.

Biological differences lead to differences in body composition between sexes. This difference begins at the beginning of life, becomes more obvious in adolescence, and remains throughout adulthood. The present study showed that sex differences existed in the vast majority of body composition indicators among Tibetan adults. According to the age- and sex-specific presentation and comparison of body composition indicators, several lines of interesting findings were revealed and subsequently discussed.

Height and weight are important indicators for evaluating human development and nutritional balance. BMI comprehensively considers human body weight and height and is frequently adopted in large-scale epidemiological studies due to its availability and relevance. It was reported that a high BMI is a major risk factor for chronic conditions such as cardiovascular disease, diabetes, musculoskeletal disorders, and some cancers (36). In this study, the average BMI value of all women in Lhasa ranged from 24.04 ± 4.02 (kg/m^2^). In the 41~ age group, it began to increase, indicating an increased risk of overweight, obesity, and related health complications. Thus, females aged 40 and above in Lhasa should be regarded as a key population for the prevention and intervention of overweight, obesity and related chronic diseases.

Obesity and other conditions related to excessive energy intake have exhibited an obvious upward trend in recent years. To formulate a reasonable reference value of energy intake, it is necessary to obtain the BMRs of different populations. The results of this study show that BMR decreases non-linearly with age, and the rate of decline accelerates with age, which is consistent with previous research (37). In the adult population in southern China, the increase in BMR was independently and negatively correlated with all-cause mortality among elderly male subjects (38); in particular, this is the first study to show that reduced BMR is an important risk factor for death among elderly individuals. In contrast, long-lived people can maintain low-energy metabolism (39–41). As such, the biological significance of BMR remains controversial and inconclusive. Regardless, our sex-, age-, and altitude-specific BMR data of Tibetan adults are of great importance for BMR-related research among the native plateau populations in the future.

FFM refers to body weight after fat removal, which is mainly related to muscle and bone. Both cross-sectional survey and follow-up research results show that the FFM of men and women remains relatively stable throughout adulthood, and the FFM of men is always higher than that of women (16, 42). This between-sex difference in FFM was further observed in our study. Janssen et al. analyzed the changes in SMM with age by magnetic resonance imaging (MRI) and found that SMM was relatively stable before 45 years of age and then began to decrease substantially with the aging process in both men and women (43). The changes in the FFM and SMM of the native plateau population with age measured by bioelectrical impedance analysis (BIA) in this study were generally in accordance with the abovementioned results. At the end of the twentieth century, the accelerated loss of skeletal muscle mass in elderly people attracted increasing attention, and Rosenberg (44) first quoted the Greek word “sarcopenia” to express this phenomenon. Our previous studies showed that the diagnostic reference value of sarcopenia in the plateau population is significantly lower than that in the plain population and that the sarcopenia incidence in the Qinghai-Tibet Plateau population was significantly higher than that in the plain population (26). On the basis of studies by others and ourselves, it is suggested that the cutoff value for sarcopenia in plateau populations should be further established based on altitude, sex, and age. In addition, more attention should be given to those vulnerable to loss of skeletal muscle mass in high-altitude areas to take timely measures to effectively prevent the occurrence of sarcopenia.

The human skeleton is involved in the dynamic process of continuous bone formation and bone absorption. In childhood and adolescence, bone mass increases with increasing age. Although the length of bone stops growing around the age of 20, there is still a stage of bone mass growth. Generally, between the ages of 20–30, the bone mineral content and bone mineral density reach the highest value, which is termed peak bone mass (PBM) (45). Studies have shown that a 10% increase in PBM at the population level would decrease the risk of developing fracture by 50% later in life (46). In our sample, it was found that the PBM of Tibetan adults generally appears after the age of 40, which may result from retarded skeletal development in a plateau environment and warrants further investigation. In addition, our data showed that the decrease in bone mass in women after 50 years of age was significantly greater than that in men, which may be related to the loss of bone minerals caused by the decrease in estrogen levels in menopausal women (47). Therefore, proper primary health care strategies and policies, such as strengthening health care awareness, developing healthy lifestyles, and appropriate hormone replacement therapy, should be established to increase the bone mass of vulnerable individuals, especially postmenopausal women, to improve their quality of life.

Body fat content is the most variable in human body composition. FM and PBF increased with age throughout adulthood, and FM and PBF were always higher in females than in males. Although FM, PBF, VFM, and SFM in the 61~ age group of Tibetan women decreased slightly, the average value was still significantly higher than those in the 20~ age group. This may be related to the fact that women enter menopause, and the change in estrogen affects fat synthesis (48). A large number of studies have confirmed that FM is closely related to the occurrence of chronic diseases. High PBF, especially high visceral fat mass, increases the risk of type II diabetes and metabolic syndrome (49). SFM and VFM are related to increases in fasting blood glucose, blood lipids, blood pressure and cardiovascular diseases (50). Sarcopenic obesity (SO) is a new type of obesity and a high-risk geriatric syndrome in elderly individuals. SO is characterized by the conjunction of insufficient muscle mass and function and excessive fat mass and is associated with many adverse health consequences, such as frailty, falls, disability, increased incidence rate and mortality, and the development of post-operative Clavien-Dindo grade ≥ II complications in women undergoing DIEP-flap breast reconstruction (51, 52). Thus, the diagnostic criteria of SO in the indigenous plateau population are worthy of further research. The results of this study suggest that menopausal women and middle-aged and elderly men are the key population for the prevention and treatment of fat-related chronic diseases. A low-calorie diet and appropriate exercise training should be encouraged to increase muscle, reduce fat, and improve physical fitness and function.

Changes in ICW and ECW embody the state of nutrient metabolism in the human body to a certain degree. Understanding the optimal fluid volume balance will facilitate the clinical application of body fluid assessment methods. A recent study revealed an association between the reduction in ECW and systolic blood pressure (SBP) with dialysis, and researchers advocated monitoring changes in ECW during dialysis to reduce the risk of intradialytic hypotension (53). This study showed that the TBW, ICW, and ECW values in females increased first and then decreased thereafter during the lifetime, with peak values appearing in the 41~ group. In contrast, among males, TBW and ICW exhibited a downward trend, and ECW exhibited an upward trend with increasing age. These results suggested that the ECW values of Tibetan adults relatively increased with age (compared with ICW and TBW), which would potentially increase the risk of hypertension, especially in males. However, due to the lack of relevant data for other populations, this phenomenon needs further exploration. Studies have shown that the decrease in cell volume due to aging, muscle attenuation and volume overload can cause fluid imbalance between the ICW and ECW, which is more obvious after the age of 70 (54, 55). In fact, the ratio of ECW to ICW is similar to that of ECW to TBW, which is related to nutritional status (55) or disease outcome (56, 57).

This study has two major limitations. First, the precision of BIA is not as high as that of dual-energy X-ray absorptiometry (DXA), which is a well-recognized approach for body composition measurement. However, BIA, which is inexpensive, easy to use, rapid and safe, seems more practical in the plateau region than the expensive and difficult to carry out DXA. Most Tibetans live in a decentralized manner, and their medical and economic conditions are poor. Second, the sample size was not large enough, especially that in the 61~ group in Shigatse. Insufficiency of sample size may lead to a certain deviation in the reference values of body composition of the plateau population.

## Conclusion

This study provides the first normative reference data of body composition for healthy indigenous individuals on the Qinghai-Tibet Plateau. These datasets are important for future research related to body composition. A considerable decrease in body composition values in >4,000 m-altitude areas suggests that body composition cutoff values should be established by altitude. Age-, sex-, and altitude-specific alterations in body composition values also inform the prevention and amendment of abnormal body composition changes and resultant health complications.

## Data availability statement

The raw data supporting the conclusions of this article will be made available by the authors, without undue reservation.

## Ethics statement

This cross-sectional study was approved by the Ethics Committee of Jinzhou Medical University (LLSC2015001) and was conducted in accordance with the Declaration of Helsinki. Written informed consent was obtained from all participants.

## Author contributions

Conceptualization and writing—review and editing: WL, HD, and YW. Data curation and investigation: WL, XL, TH, YC, and HZ. Formal analysis, methodology, and writing—original draft preparation: WL and HD. Funding acquisition and resources: YW. Project administration: HD. Software and validation: HZ. Supervision: YW and HD. Visualization: TH. All authors have read and agreed to the published version of the manuscript.

## Funding

This work was supported by the National Natural Science Foundation of China (Grant No. 31571233).

## Conflict of interest

The authors declare that the research was conducted in the absence of any commercial or financial relationships that could be construed as a potential conflict of interest.

## Publisher's note

All claims expressed in this article are solely those of the authors and do not necessarily represent those of their affiliated organizations, or those of the publisher, the editors and the reviewers. Any product that may be evaluated in this article, or claim that may be made by its manufacturer, is not guaranteed or endorsed by the publisher.
